# Indicating spinal joint mobilisations or manipulations in patients with neck or low-back pain: protocol of an inter-examiner reliability study among manual therapists

**DOI:** 10.1186/2045-709X-22-22

**Published:** 2014-06-20

**Authors:** Emiel van Trijffel, Robert Lindeboom, Patrick MM Bossuyt, Maarten A Schmitt, Cees Lucas, Bart W Koes, Rob AB Oostendorp

**Affiliations:** 1 Department of Clinical Epidemiology, Biostatistics & Bioinformatics, Academic Medical Centre, University of Amsterdam, Amsterdam, the Netherlands; 2 Institute for Master Education in Musculoskeletal Therapy, Amersfoort, the Netherlands; 3 Department of General Practice, Erasmus MC University Medical Centre, Rotterdam, the Netherlands; 4 Scientific Institute for Quality of Healthcare, Radboud University Nijmegen Medical Centre, Nijmegen, the Netherlands; 5 Department of Rehabilitation, Physiotherapy and Manual Therapy, Faculty of Medicine and Pharmacology, Free University of Brussels, Brussels, Belgium

**Keywords:** Manual therapy, Motion assessment, Diagnostics, Decision-making, Reliability, Clinical reasoning, Neck pain, Back pain

## Abstract

**Background:**

Manual spinal joint mobilisations and manipulations are widely used treatments in patients with neck and low-back pain. Inter-examiner reliability of passive intervertebral motion assessment of the cervical and lumbar spine, perceived as important for indicating these interventions, is poor within a univariable approach. The diagnostic process as a whole in daily practice in manual therapy has a multivariable character, however, in which the use and interpretation of passive intervertebral motion assessment depend on earlier results from the diagnostic process. To date, the inter-examiner reliability among manual therapists of a multivariable diagnostic decision-making process in patients with neck or low-back pain is unknown.

**Methods:**

This study will be conducted as a repeated-measures design in which 14 pairs of manual therapists independently examine a consecutive series of a planned total of 165 patients with neck or low-back pain presenting in primary care physiotherapy. Primary outcome measure is therapists’ decision about whether or not manual spinal joint mobilisations or manipulations, or both, are indicated in each patient, alone or as part of a multimodal treatment. Therapists will largely be free to conduct the full diagnostic process based on their formulated examination objectives. For each pair of therapists, 2×2 tables will be constructed and reliability for the dichotomous decision will be expressed using Cohen’s kappa. In addition, observed agreement, prevalence of positive decisions, prevalence index, bias index, and specific agreement in positive and negative decisions will be calculated. Univariable logistic regression analysis of concordant decisions will be performed to explore which demographic, professional, or clinical factors contributed to reliability.

**Discussion:**

This study will provide an estimate of the inter-examiner reliability among manual therapists of indicating spinal joint mobilisations or manipulations in patients with neck or low-back pain based on a multivariable diagnostic reasoning and decision-making process, as opposed to reliability of individual tests. As such, it is proposed as an initial step toward the development of an alternative approach to current classification systems and prediction rules for identifying those patients with spinal disorders that may show a better response to manual therapy which can be incorporated in randomised clinical trials. Potential methodological limitations of this study are discussed.

## Background

Neck and low-back pain are common and costly disorders in adult general populations [1-6]. Manual spinal joint mobilisations and manipulations are widely used treatments in patients with these complaints [7,8]. Although the underlying mechanisms of these treatments are far from understood, spinal joint mobilisations and manipulations are effective as well as cost-effective in patients with non-specific neck and low-back pain although no more effective than other treatment modalities [9-14].

Traditionally, manual therapy has a strong focus on the diagnostics, treatment, and evaluation of spinal joint function by emphasising the use of passive physiological and accessory movements [15-17]. Passive intervertebral motion (PIVM) assessment is used to judge the quantity and quality of functions of spinal motion segments and is assumed to play an important role in diagnostically classifying patients and selecting treatment [18]. Dutch, New Zealand, and USA manual therapists indeed believe that passive spinal mobility testing is important for deciding on manual mobilisation or manipulation as a treatment option [19,20]. Moreover, a recent international, multidisciplinary survey showed that PIVM assessment is the most commonly used impairment outcome measure in patients with neck pain [21].

In order to yield accurate and uniform decisions about treatment options for patients, test results need to be reliable [22]. Reliability is a component of reproducibility along with agreement and reflects the extent to which test results can diagnostically discriminate between patients despite measurement errors [23,24]. Agreement, on the other hand, concerns the possibility of examiners to obtain the same test results on different measurement occasions [25]. Systematic reviews have consistently shown poor inter-examiner reliability for spinal physical tests, and for PIVM assessment in particular [26-30]. However, the large majority of studies investigating the reliability of physical tests and PIVM assessment can be regarded as test research following a single-test or univariable approach, thus neglecting the multivariable character of the diagnostic process as opposed to diagnostic research [31].

Physiotherapists conduct a diagnostic process by collecting data through interview and physical examination and by generating hypotheses as to why a problem exists in order to reach a decision about appropriate patient management [32,33]. During this diagnostic process, manual therapists indeed seem to apply, amongst others, a hypothetico-deductive way of clinical reasoning [34,35]. PIVM assessment is usually conducted after history-taking, questionnaires, and other physical tests and is indicated after interpreting earlier clinical information and formulating specific hypotheses about spinal joint dysfunction [35]. Moreover, Canadian manual therapists reported to decide on manual mobilisation or manipulation based on their whole clinical assessment and clinical reasoning in a patient [36]. It is therefore reasonable to assume that the diagnostic process in manual therapy has a multivariable character.

Over the last three decades, many systems have been developed for classifying patients with spinal disorders, in particular for those with low-back pain [37]. A systematic review found 28 systems for classifying chronic low-back pain alone and it was concluded that there was insufficient evidence to support or recommend any particular system for use in clinical description, determining prognosis, or predicting response to treatment [38]. Some systems were tested for their inter-examiner reliability, but evidence was either conflicting or moderate to strong for poor reliability [27]. On the other hand, using clusters of tests for diagnosing sacroiliac joint dysfunction yielded acceptable reliability [39-41]. However, the majority of these systems either lack evidence for their reliability, only use certain parts of the clinical examination (e.g. only physical tests), are prescriptive in their application, do not include PIVM assessment, are not related to manual therapy interventions, or do not direct towards treatment decisions. Some systems [42,43] were developed as treatment-based classification algorithms for subgrouping patients with low-back pain and were strongly based on factors derived from several clinical prediction rules [44-47]. However, these rules lack validation, and methodological and statistical issues regarding their development have been raised [48]. In contrast to the field of classification systems for low-back pain, the development and number of systems for classifying neck pain patients lie far behind. Besides a treatment-based classification system for physiotherapy interventions [49], clinical prediction rules have been derived to identify factors that predict response to spinal manipulation in patients with neck pain but with identical problems as in the rules for low-back pain as mentioned above [50-55]. In a systematic review, Gemmell and Miller [56] found poor inter-examiner reliability of multitest regimens using only physical tests for identifying manipulable spinal lesions in chiropractic. Including pain scores and medical history next to manual examination of spinal motion segments resulted in high accuracy in identifying neck pain patients [57]. To summarise, however, the value of the diagnostic process as a whole to classify patients with neck or low-back pain in order to decide whether or not spinal mobilisations or manipulations are indicated remains unclear.

This is the protocol of a study that aims to determine the inter-examiner reliability among Dutch manual therapists of indicating spinal joint mobilisations or manipulations in patients with neck or low-back pain based on a multivariable, hypothesis-based diagnostic reasoning and decision-making process. Secondly, using univariable logistic regression analysis of concordant decisions about indications, we will explore which demographic, professional, and clinical factors can explain variation in reliability of therapists’ decisions with specific attention to the contribution of PIVM assessment.

## Methods

### Design

This study will be conducted as a repeated-measures design in which pairs of manual therapists independently examine a consecutive series of patients with neck or low-back pain presenting in primary care physiotherapy in the Netherlands. Primary outcome measure is therapists’ decision about whether or not spinal manual therapy (SMT) is indicated in each patient, alone or as part of a multimodal treatment. SMT is defined here as either spinal joint mobilisations or manipulations, or both. Therapists will largely be free to conduct the full diagnostic process as they are routinely used to.

### Participants

Consecutive patients aged 18 years or older presenting with a primary complaint of neck or low-back pain, either referred to primary care physiotherapy by their general practitioner or medical specialist, or by self-referral, will be eligible for participation in the study. Neck pain is defined as pain in the region between the superior nuchal line, the external occipital protuberance, the spines of the scapula, the superior border of the clavicula, and the suprasternal notch, with or without radiation to the head, trunk, or upper limbs [58]. Patients will not be eligible when headache or dizziness is their dominant complaint. Low-back pain is defined as pain or discomfort localised below the costal margin and above the inferior gluteal folds, with or without radiation to the lower limbs [59]. All patients who are assumed to have non-specific or (non-serious) specific neck or low-back pain with a potential indication for SMT will be included. Patients who are not able to speak or read Dutch fluently will be ineligible. Patients will receive verbal and written information on all aspects of the study and will be asked to provide written consent at their inclusion. The Central Committee for Research involving Human Subjects (CCMO, the Hague, the Netherlands) decided that a full evaluation of the study protocol by a medical ethical committee was not required because patients will undergo a diagnostic process similar to routine daily practice.

### Examiners

Examiners will be manual therapists working at least 20 hours a week in their private practices in the Netherlands and registered by the Dutch Association for Manual Therapy or the Royal Dutch Society for Physical Therapy. From a database of those graduated from the Institute for Master Education in Musculoskeletal Therapy (SOMT: Stichting Opleidingen Musculoskeletale Therapie, Amersfoort, the Netherlands), 14 pairs of manual therapists will be invited to participate. Each pair works together in the same practice and practices will be selected based on their ability to logistically organise the study. We aim to include therapists who vary in years of clinical experience in manual therapy. Therapists will attend an information session followed by a two-hour training session in which procedures for digitally registering data are explained and practised. They will not receive additional training in history-taking, physical examination procedures, or using questionnaires. Pairs of therapists will be strictly requested not to discuss their experiences during the study with each other until their last patient has been included. Gender, age, years of clinical experience in manual therapy, highest diploma, practice setting, weekly amount of work related to spinal disorders (hours), teaching experience (yes/no), and participation in research (yes/no) will be recorded as professional characteristics from the participating therapists.

In each practice, a third colleague will function as a research assistant to coordinate the inclusion and flow of patients. Research assistants will be instructed with respect to applying the inclusion criteria, the order of assigning patients to therapists, and assuring blinding procedures.

### Procedures

From eligible patients, demographic (gender, age, marital status, working status) and clinical (type of complaints (neck or low-back pain), duration of complaints (days), radiation (yes/no), traumatic origin (yes/no), comorbidity (yes/no)) data will be recorded as baseline data by the local research assistant. In addition, baseline pain and disability will be determined using the Numeric Pain Rating Scale (NPRS 0–10, higher scores indicate higher pain intensity), and the Quebec Back Pain Disability Scale (QBPDS 0–100, higher scores indicate higher disability) for low-back pain patients and the Neck Disability Index Dutch Language Version (NDI-DLV 0–50, higher scores indicate higher pain and disability) for neck pain patients, respectively. The NPRS is a reliable and valid scale to measure pain intensity in adults [60]. The Dutch version of the QBPDS is a reliable and valid instrument for measuring disability in low-back pain patients [61] and the Dutch version of the NDI is recommended for measuring pain and disability in patients with neck pain [62].

All baseline data will be available to each therapist before he or she starts the diagnostic process. The first therapist of each pair will be the treating therapist to whom the patient was assigned to, so the order in which both therapists act as the first examiner will vary according to the practice’s planning. The first therapist will screen all consecutive patients with neck or low-back pain for the presence of red flags [63]. In accordance with guidelines in the Netherlands [64], patients suspected of having serious (spinal or non-spinal) pathology will not enter the study which will be recorded. Patients will then undergo a full history-taking by the first therapist. The therapist will record his or her findings as well as proposed hypotheses about patient’s health status by formulating explicit objectives for further examination. The therapist will then choose the diagnostic procedures (e.g. observation, physical tests, performance tests, questionnaires) that he or she plans to perform in the patient. After performing each procedure, its outcome will be recorded. If PIVM assessment is indicated, therapists will use three-dimensional coupled movements in flexion and extension directions for each individual motion segment [65]. Movements will be judged on mobility (hypermobile-normal-hypomobile), resistance perceived by the therapist during the movement (increased resistance or stiffness yes/no), resistance perceived by the therapist at the end of the movement (end-feel) (increased resistance or stiffness at the end of the movement yes/no), and pain provocation (yes/no). Therapists will perform a maximum of three repetitions for each movement per direction per spinal motion segment to afford the best stiffness discriminability [66].

The therapist will then be asked to record whether he or she has made any changes to the original examination objectives as well as to specify these changes, and a diagnostic conclusion in terms of specific or non-specific neck or low-back pain is given. Finally, the therapist will make the decision about whether or not SMT is indicated in the patient and, when indicated, it will also be stated whether mobilisations or manipulations, or both, are indicated, and to which spinal motion segments these techniques would be targeted. In addition, the therapist will rate his or her level of certainty of the primary decision about the indication on a bipolar seven-point scale ranging from -3 (completely uncertain) to 3 (completely certain). It will also be recorded which other interventions he or she believes would further be indicated in the patient. However, at this point, no actual treatment will be provided.

After the first therapist has performed the full examination, he or she will leave the examination room and the patient will be given a 10 minute break. After checking whether all data have been registered, the research assistant then guides the second therapist into the room and makes sure that there is no visual or verbal contact between the two therapists. The second therapist will then conduct the full diagnostic process, excluding the screening for red flags, whilst being unaware of the outcomes of the first examination. Patients will be requested not to mention any outcomes or conclusions from the first examination. Both therapists will record all their findings and data into a fit-for-purpose software program. The research assistant will check whether all data have been entered by both therapists.

### Statistical analysis

Demographic and clinical baseline characteristics of patients will be summarised using descriptive statistics. Absolute and relative frequencies are used to describe categorical data. Ordinal data relating to patients’ pain and disability will be described with their median and interquartile range. Normally distributed numerical data will be summarised by their mean and standard deviation. In case of non-normality, median and interquartile range are presented. Examination objectives as formulated by therapists will be classified by one researcher (EvT) according to the framework of the World Health Organization’s *International Classification of Functioning, Disability and Health* (ICF) [67] to describe patients’ functioning in terms of impairments of neuromusculoskeletal and movement-related functions, activity limitations and participation restrictions, and personal and environmental factors. Diagnostic procedures will be listed and described with their frequencies, and also outcomes of PIVM assessment, changes to the original examination objectives, diagnostic conclusions, and examiners’ level of certainty of their decision about the treatment indication will be summarised. Concordance between the formulated examination objectives concerning spinal joint motion function and the actual use of PIVM assessment will be presented as frequencies.

For each pair of therapists, 2×2 tables will be constructed and reliability for the dichotomous positive or negative decisions about whether or not SMT is indicated will be calculated as chance-corrected reliability using Cohen’s kappa [68]. As recommended by Cicchetti and Feinstein [69] and Byrt et al. [70], observed agreement (%), prevalence of positive decisions (mobilisations and/or manipulations indicated) relative to the total number of indications, prevalence index (PI), bias index (BI), and specific agreement (%) in positive (p_pos_) and negative (p_neg_) decisions will be calculated in order to evaluate whether kappa was influenced by high prevalence of positive or negative decisions, or by systematic bias between examiners. PI reflects the difference between the proportion of agreement on positive indications as compared to that of negative indications. PI ranges between 0 and 1, and is high when the prevalence of concordant positive (or negative) indications is high, chance agreement is consequently also high, and kappa is reduced accordingly (prevalence effect) [71]. BI provides a quantification of the extent to which examiners disagree on the proportions of positive (or negative) indications. BI also ranges between 0 and 1, and is high when the difference between the discordant indications is high, chance agreement is consequently low, and kappa is inflated accordingly (bias) [71]. P_pos_ and P_neg_ are the proportions of agreement on positive and negative indications, respectively, relative to the total number of positive and negative indications, respectively, from both therapists. Overall kappa (95% CI) will be calculated as a generalized chance-corrected reliability across all pairs of therapists. See Additional file 1 for formulas.

In addition, for each pair of therapists, separate 2×2 tables will be presented for judgements about the indication for PIVM assessment and for judgements about mobility, end-feel, and pain provocation obtained from PIVM assessment (four tables in total). Observed agreement, prevalence of positive decisions, PI, BI, p_pos_, p_neg_, and overall kappa (95% CI) will also be calculated. Analyses will be conducted using DAG_Stat [72].

Kappa (95% CI) is interpreted in accordance with value labels as assigned by Landis & Koch [73]: <0.00: poor, 0.00-0.20: slight, 0.21-0.40: fair, 0.41-0.60: moderate, 0.61-0.80: substantial, 0.81-1.00: almost perfect. We arbitrarily assume a lower bound of the 95% CI around overall kappa of 0.60 to indicate acceptable reliability.

Univariable logistic regression analysis will be performed to explore which demographic, professional, and clinical factors contributed to the reliability of therapists’ decision-making. Firstly, patients’ demographic and clinical factors at baseline will concern their gender, age, type of complaints, duration of complaints (less or more than three months), radiation, traumatic origin, comorbidity, pain intensity, and disability. Such factors are associated with variation in diagnostic accuracy [74], but evidence in the context of reliability is lacking. Secondly, therapists’ professional factors will include their clinical experience and weekly amount of work related to spinal disorders. Weekly amount of work related to spinal disorders was positively associated with perceived importance and confidence related to the use and interpretation of PIVM assessment [20] and may, therefore, contribute to variation in diagnostic decision-making. In addition, other clinical factors will be explored involving PIVM assessment (indicated or not, and judgements on mobility, resistance, and pain provocation), the diagnostic conclusion (specific or non-specific neck or low-back pain), therapists’ level of certainty of their decision about the treatment indication, and the concordance between examination objectives and the use of PIVM assessment. Factors will be entered in the model as single covariates with the concordant decisions, either positive or negative, as the dependent variable. Concordant decisions will be coded as 1 while the discordant decisions will be coded 0. Therapists’ experience and work related to spinal disorders will be entered as mean scores from each pair. A p-value <0.05 indicates a statistically significant association between a factor and a concordant decision about whether or not SMT is indicated.

With a sample size of 165, a two-sided 95% CI around kappa would extend ±0.109 from the observed value of kappa, assuming a true value of kappa of 0.70, and a prevalence of positive decisions of 50%. Consequently, each pair of examiners will be asked to include 12 patients. Multiple imputation will be used to handle records with data points missing at random. If, for any reason, data on the primary outcome measure are not available or obtainable from one or both therapists, all data from this patient will be excluded from the analysis and the pair of therapists will be asked to include a new patient. Analyses will be conducted using IBM SPSS Statistics for Windows version 22.

## Discussion

The results of this study will provide 1) an estimate of the inter-examiner reliability among manual therapists of indicating SMT in patients with neck or low-back pain based on a multivariable diagnostic reasoning and decision-making process, as opposed to reliability of individual clinical tests, and 2) a first exploration of which demographic, professional, or clinical factors can explain variation in the reliability of therapists’ decision-making with specific attention to the contribution of PIVM assessment. We do not aim or hypothesise that reliability from a multivariable approach to clinical diagnostics will be higher than that from individual test diagnostics. Rather, we believe that such an estimate will be a more real resemblance of the reliability among therapists of making decisions in daily practice concerning the distinction between patients who are indicated for SMT and those who are not. In addition, this approach will add to the ongoing discussion of the identification of specific subgroups of patients that may be more likely to respond to SMT and we propose alternative research strategies for establishing treatment effects.

It has been recognised that treatment effects of SMT, or any other physiotherapy modality for that matter, especially in patients with low-back pain, are, on average, small which may be due to heterogeneity of patients obscuring a wide range of individual treatment responses and variation of treatment effects [75]. Ever since the mid-nineties of the last century, identifying subgroups of patients that may benefit more from specific or targeted interventions has had the highest research priority [76-81]. As a result, there has been a proliferation of subgrouping systems aiming to identify people with a particular pathoanatomical condition, a particular prognosis, or those that are more likely to respond favorably to treatment [82]. Primary care clinicians themselves do not believe that low-back pain is one condition and they treat patients differently based on patterns of clinical signs and symptoms [83]. Moreover, they classify patients predominantly based on pathoanatomy, but they show little consensus regarding these related patterns [84]. With the aim to identify patients that may be more likely to show a positive response to spinal manipulation, clinical prediction rules have been derived to identify predictors in patients with neck and low-back pain [44-47,50-55]. Unfortunately, systematic reviews have consistently concluded that there is, as yet, insufficient evidence to support the general application of these rules [85-89]. Another systematic review found significant treatment effects favoring subgroup-specific SMT over a number of comparison treatments for pain and disability at short and intermediate follow-up based on low-quality trials [90]. Foster et al. [75] concluded that no subgrouping approaches have yet passed the tests for clinical value and robustness of evidence, and there is still a long way to go before closer matching of treatments to patient characteristics becomes a clinical reality. Indeed, two decades after the derivation of the Ottawa Ankle Rules [91], their validation and implementation is still an ongoing research process worldwide and it can be assumed that following a similar pathway for far more complex problems such as the treatment of non-specific neck and low-back pain may be even more time-consuming.

When determining treatment effects of SMT, randomised clinical trials currently do not make use of patients’ full clinical health profile according to the domains of the ICF for targeting treatment. For instance, Cochrane Reviews consider primary studies including participants only based on their age and the presence of pain with or without radiation [11,13,14]. The resulting heterogeneity among trial participants and the subsequent dilution of treatment effects may be deleterious to SMT as its effectiveness may be underestimated for certain groups of patients. The majority of primary studies in patients with neck pain do not apply well-defined clinical criteria to select patients for SMT and if they do, they use only one physical test, such as a mobility test or a pain provocation test, in order to diagnose neck pain from a mechanical origin [92]. It is stated that clinical tests are not valid or reliable to allow targeting treatment in clinical trials [84]. This is certainly true when the reliability of individual physical tests is considered [26-30]. However, several of the increasingly popular predication rules also contain clinical variables that are unreliable, including PIVM assessment [42,46,88]. Targeting SMT to a more homogeneous group of patients with neck or low-back pain, based on a multivariable diagnostic process resembling daily practice, may outweigh the disadvantages of the current selection procedures in randomised clinical trials.

**Figure 1:**
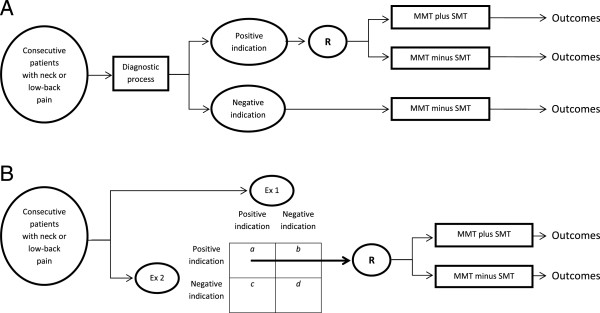
**A. Design of an RCT including patients positively indicated for SMT when lower bound of 95% CI around kappa >0.60 and BI <0.10. B**. Design of an RCT including only patients positively indicated for SMT by two examiners when kappa <0.60 but p_pos_ (or p_neg_) >60%.

Awaiting evidence from the further validation of prediction rules and other classification systems, our study could offer an initial step toward a faster and easier development of an alternative approach to the identification of those patients with spinal disorders that may show a better response to SMT based on a multivariable decision process. A satisfactory level of reliability is a prerequisite for incorporating such decision-making into the design of randomised clinical trials for establishing treatment effects of SMT and thereby validating the approach. When reliability (lower bound of 95% CI around kappa) exceeds 0.60 and with BI, arbitrarily, <0.10, patients with neck or low-back pain with a positive indication can be randomised to receive, for instance, either manual mobilisations or manipulations, or both, within a multimodal treatment on the one hand or multimodal treatment without mobilisations or manipulations on the other (Figure 1A). Should reliability be below this cut-off but with p_pos_ (or p_neg_), arbitrarily, >60%, this strategy can still be used by randomising only those patients of which the indication was agreed upon by two manual therapists (Figure 1B). P_pos_ and p_neg_ here indicate the absolute specific agreement on positive or negative indications, respectively, between therapists [25].

With respect to our second research objective, it is important to note that empirical evidence for sources of bias and variation in reliability studies is lacking contrary to studies of diagnostic accuracy [74,93-95]. Variation arises from differences between studies, for example, in terms of demographic and disease features of study participants, characteristics of examiners, setting, or test protocol. As such, it does not lead to biased estimates of reliability, but it can limit the applicability of study results [94]. Knowledge of factors that explain variation in reliability may inform ways to improve reliability. For instance, examiner training and choosing a group of more heterogeneous study participants have been mentioned as improvement strategies, but both have their limitations and lack supporting evidence [24]. Systematic reviews may reveal subgroups of participants, examiners, or tests that consistently show higher or lower reliability. In systematic reviews, between-study comparisons are conducted to search for these subgroups as sources of variation. However, these comparisons are less valid as they are hampered by the often strong clinical and methodological heterogeneity between studies [96]. In addition, the identification of these sources of variation becomes even more troublesome when reliability is consistently low (or high) across studies. Within-study comparisons are the preferred method to explore variation in reliability. To date, very few studies have been undertaken in the field of manual therapy with this aim and method. Cook et al. [97] investigated factors related to the large variability of forces used during passive accessory intervertebral movements and they found that examiners’ age, gender, experience, background and education, and frequency of use did not contribute to this variation. We present simple logistic regression analysis of concordant decisions as a flexible method that can easily be incorporated in any reliability study to explore and explain variation in reliability from a large number of demographic, professional, and clinical factors.

### Potential limitations of this study

This study protocol presents several new approaches to investigating and analysing decision-making in manual therapy and to reliability research in general. Several of its methods need further discussion in order to appraise their effect on the validity and generalisability of the study’s results. First, establishing examination objectives for physical examination by physiotherapists has been used in earlier studies [98,99]. However, the prospective formulation and registration of examination objectives is far from common practice for physiotherapists in the Netherlands [100]. The specific training of our examiners in the formulation and digital registration of these objectives may diminish the generalisability of the estimated reliability of indicating SMT. We encourage that establishing and prospectively registering of examination objectives become an integral part of daily practice in physiotherapy.

Stability of participants’ characteristics is a prerequisite for the valid estimation of reliability [23]. However, very few empirical data are available as to the minimum length of the time period between test procedures that ensures that patients’ responses to questions and physical tests, such as joint motion assessment, will remain unchanged. Shirley et al. [101] reported that stiffness responses to repeated mechanical posteroanterior loading of lumbar motion segments returned to the pre-testing state within five minutes. On the other hand, a 30-minute recovery period after 30 minutes of *in vitro* creep loading of the lumbar spine was not sufficient to return to the baseline situation [102]. By incorporating a 10 minute break for patients between examinations and limiting the number of movement repetitions during PIVM assessment, we are more confident that underestimation of reliability will be avoided. Research into the natural variation over time within and between individuals regarding joint mobility and other body functions, as well as into the variation induced by the physical examination itself, is needed.

Our sample size calculation strongly depends on the assumed prevalence of positive indications which was based on data from the numerous studies on practice patterns among physiotherapists in the treatment of patients with neck and low back pain [103-113]. Within the large variation in choices of treatment options by therapists, mobilisations and manipulations were only rarely among the most preferred options and their frequency of use ranged from 16% to 83% and from 2% to 37%, respectively. These figures were not substantially different for specific subgroups of manual therapists who reported remarkably low frequencies of use of manipulations in the cervical region [36,114-116]. As we will consider reliability of indicating either mobilisations or manipulations, or both, we assume a 50% prevalence of positive indications. Choosing a higher or lower prevalence would have resulted in a larger required sample [117].

In our sample of manual therapists and patients, we cannot rule out the possibility of a substantially higher (or lower) prevalence of positive indications for SMT. Because of such a skewed distribution of decisions, a distorted interpretation of kappa could then occur. Recently, kappa, as a relative measure of reliability, has been criticised because it can only provide information about the ability to distinguish between patients on a sample level [25]. The authors suggest using the specific agreement parameters (p_pos_ and p_neg_) as absolute measures to quantify observer variation regarding a certain diagnosis or decision on an individual patient level [25]. No single omnibus index, however, can be satisfactory for all purpose and situations [69,70]. Therefore, we will calculate all recommended parameters from the 2×2 tables to allow full interpretation of reliability and agreement as related to the prevalence of concordant and discordant indications. We will not, however, correct kappa for prevalence effects and bias, for instance by calculating prevalence-adjusted bias-adjusted kappa, because this would generate values of reliability that no longer relate to the original situation [117,118].

We will select pairs of manual therapists as examiners that share a common educational background. With this background from the largest institute for manual therapy education in the Netherlands, they likely form a representative sample from the Dutch population of manual therapists registered with the Dutch Association for Manual Therapy or the Royal Dutch Society for Physical Therapy. Manual therapy education in the Netherlands is strongly embedded within international concepts. In these traditional concepts, especially passive joint motion assessment takes a prominent place [15]. Therefore, we suppose that the results of this study will to a certain extent be generalisable to populations of manual therapists outside the Netherlands. We do, however, suggest that this study be replicated over different countries and concepts to account for local idiosyncrasies in clinical reasoning and decision-making. In addition, for practical reasons, we will choose pairs of manual therapists that work in the same practice. This may inflate reliability and by pairing therapists with different levels of experience, we aim to minimise this potential threat to the validity of the study.

Finally, when analysing the reliability of indicating SMT, we will not distinguish specifically between mobilisations or manipulations. Despite the disparate mechanisms of these interventions [9,119], no evidence is available on whether one or the other, or both, should be preferred in any clinical situation. Results of randomised controlled trials have been conflicting so far [120-123]. New research should focus on the relationship between clinical findings, the choice for either mobilisation or manipulation, and subsequent clinical outcomes.

## Abbreviations

ICF: International Classification of Functioning, Disability and Health; PIVM: Passive intervertebral motion; SMT: Spinal manual therapy.

## Competing interests

The authors declare that they have no competing interests.

## Authors’ contributions

EvT is the principal investigator of the study, developed the research questions and methods, obtained ethical approval, and drafted the article. RL, MS, CL, BK, RO assisted in the development of the methods and wrote the study protocol. RL, EvT, PB developed the statistical plan for this protocol. PB, RO supervised the project. All authors assisted with revisions to the study protocol and methods, and approved the final version of the article.

## Supplementary Material

Additional file 1 Formulas for kappa and associated measures. Click here for file
