# Supplementary material for: Indicating spinal joint mobilisations or manipulations in patients with neck or low-back pain: protocol of an inter-examiner reliability study among manual therapists
Source: Chiropr Man Therap. 2014 Jun 20;22:22. doi: 10.1186/2045-709X-22-22 (PMC4074830; doi:10.1186/2045-709X-22-22)
Supplement: Additional file 1 — Formulas for kappa and associated measures. [file 2045-709X-22-22-S1.doc]

**Additional file 1**

Formulas

|  |  |  | *Examiner 1* | |  |
| --- | --- | --- | --- | --- | --- |
|  |  |  | **Indication** | |  |
|  |  |  | Positive | Negative |  |
| *Examiner 2* | **Indication** | Positive | *a* | *b* | *g_1_* |
|  |  | Negative | *c* | *d* | *g_2_* |
|  |  |  | *f_1_* | *f_2_* | *n* |


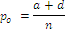


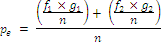


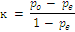


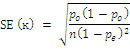


95% CI (ĸ) = ĸ – 1.96 x SE (ĸ) to ĸ + 1.96 x SE (ĸ)


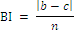


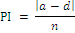


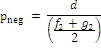


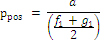


BI: bias index, CI: confidence interval, ĸ: kappa, p_e_: expected agreement by chance, PI: prevalence index, p_neg_: proportion of agreement on negative indications, p_o_: observed agreement, p_pos_: proportion of agreement on positive indications, SE: standard error
